# Influence of magnetic field on barium sulfate incrustation from aqueous solutions

**DOI:** 10.1016/j.heliyon.2019.e02032

**Published:** 2019-07-05

**Authors:** Zurel S. Costa, Cristiano T. Meneses, Bruno Castro, Fabiane S. Serpa, Elton Franceschi, Gustavo R. Borges, Cláudio Dariva, Giancarlo R. Salazar-Banda

**Affiliations:** a NUESC/LEN/ITP, Núcleo de Estudos em Sistemas Coloidais, PEP/PBI/UNIT, Universidade Tiradentes, Av. Murilo Dantas, 300, Aracaju, SE 49032-490, Brazil; b Universidade Federal de Sergipe, Campus de Itabaiana, Av. Vereador Olimpio Grande, Itabaiana, SE CEP 49100-000, Brazil; c PETROBRAS/CENPES, Av. Horacio de Macedo, 950, Cidade Universitária, Rio de Janeiro, 21941-915, Rio de Janeiro (RJ), Brazil

**Keywords:** Chemical engineering, Industrial engineering, Petroleum engineering, Thermodynamics, Physical chemistry, Scales, Barium sulfate, Magnetic field, Dynamic pressure drop test

## Abstract

The formation of scales in the petroleum industry, such as those composed of calcium and barium sulfates, may reduce productivity since these sediments can partially or totally obstruct the pipes. The mitigation of these inorganic precipitates can be accomplished by using scale inhibitors or by non-intrusive physical technologies. Here, we investigated the influence of magnetic field on the incrustations of barium sulfate by analyzing the concentration of barium and sulfate ions, the solution flow rate, the capillary tube geometry, and the magnetic field intensity in a homemade experimental unit supported on the monitoring of the dynamic differential pressure. The results show that the saline concentration and the flow rate of the solutions and the geometry of the capillary tube have a significant influence on the dynamics of barium sulfate incrustation. The presence of the magnetic field tends to prolong the induction time of the barium sulfate precipitation. A semi-empirical model was used to describe the effect of the studied variables on the barium sulfate incrustation behavior. The X-ray diffraction data of the precipitated particles analyzed using the Rietveld method suggest that the use of the magnetic field favor the formation of more crystalline particles and with smaller crystallite size than those formed in the absence of a magnetic field. Optical and scanning electron microscopy measurements also corroborate with these findings. The results from this study suggest that magnetic fields can be of interest in practical crystallization processes of barium sulfate and successfully applied to decrease the speed of barium sulfate incrustation in pipelines.

## 1 Introduction

The formation of scales is an important concern in the petroleum industry. Scales occurrence can decrease the productivity due to the total or partial obstruction of the pipes, stopping equipment and blocking sedimentary rocks pores in reservoirs (Zendehboudi et al., 2014; Khormali et al., 2018). Oil industry and academy have investigated new technologies and tools to prevent and control scales formation in reservoirs, production columns, and equipment. In petroleum fields, the scales contain mainly barium sulfate, BaSO_4_, strontium sulfate, SrSO_4_, calcium sulfate, CaSO_4_, iron carbonate, FeCO_3_ and calcium carbonate, CaCO_3_ (Reis et al., 2011; Vazirian et al., 2016).

Sulfate scales are generally a consequence of the chemical incompatibility between the water naturally present in the rocks that contain the oil and gas and the injection seawater (sulfate-rich) that is used for offshore oil recovery (BinMerdhah et al., 2010; Jordan et al., 2014; Bezerra et al., 2013; Vazirian et al., 2016; Khormali et al., 2018). Also, the change in the process exploration conditions can contribute to disturbing the equilibrium conditions of brines. In this context, the knowledge of the concentration of dissociated salts in the aqueous phase at different conditions of supersaturation, pH, temperature, and pressure allows for the monitoring of the nucleation phenomena and crystal growth kinetics of these salts. The nucleation, precipitation, and growth of inorganic crystals responsible for the incrustation can be avoided/delayed using data associated with salt precipitation rates in the presence of chemicals scale inhibitors (Antony et al., 2011; Fan et al., 2009; Rosa et al., 2016). Besides the efficiency of the scale inhibitors used, the understanding of their action mechanisms is essential to reduce environmental impacts resulting from their incorrect disposal and also to minimize cost (Reis et al., 2011). For instance, Kelland (2011) used a dynamic differential pressure unit, to investigate the effect of iron ions (bivalent and trivalent) on the formation of CaCO_3_ and BaSO_4_ incrustations, in the presence and absence of their respective scale inhibitors. They concluded that the addition of 25 ppm of iron (II) has a negligible effect on the induction time of CaCO_3_ and BaSO_4_ incrustation.

On the other hand, the use of physical techniques, such as ultrasonic (Kim and Suslick, 2018) or external magnetic fields (Al Helal et al., 2018), has also been investigated to prevent the formation of scales. Studies on the application of magnetic fields in the prevention of scales have been mainly focused on the prevention of CaCO_3_ formation and its further incrustation (Cefalas et al., 2008; Sohaili et al., 2016; Chang and Tai, 2010; Kozic et al., 2010; Al Helal et al., 2019). The main effect suggested is the acceleration of the nucleation process resulting in a larger number of the formed nuclei, thus decreasing the particle size of the precipitates (Chibowski and Szcześ, 2018). In addition, changes in the crystalline structure have also been observed (Chang and Tai, 2010; Wang et al., 2012). Simonic and Urbancl (2017) observed that applying a 14400 G magnetic field favors the formation of CaCO_3_ as aragonite, whereas mainly calcite is obtained without the use of magnetic fields.

Saksono et al. (2009) investigated the influence of magnetic fields on the CaCO_3_ precipitation in solutions with high and low supersaturation. In supersaturated solution, the magnetic field strengthens ion interactions during circulation in a fluid flow system, thus reducing the CaCO_3_ precipitation. Jiang et al. (2013) evaluated the influence of the magnetic field intensity (**B**) and verified that the magnetic field modified the induction time of the CaCO_3_ incrustation under the evaluated conditions. A delay in the incrustation induction time when the fluid velocity (**V**) was 1.2 m s^−1^ with the perpendicular magnetic field of 7000 G was verified. The authors suggested that the effect of the magnetic field would be obtained for a **B**x**V** product of 8.400 Gauss m s^−1^.

Nevertheless, Silva et al. (2015) evaluated the effect of the magnetic field applied in the cations solution of the BaSO_4_ salt. They found that the BaSO_4_ incrustation can be controlled by the treatment of the electrolytic solutions with a magnetic field causing a reduction of the particle size. Moreover, evidence of the positive action of the magnetic field in the prevention of BaSO_4_ scales in onshore production units of Carmópolis (Sergipe, Brazil) has also been reported. However, it is still necessary to conduct comprehensive and systematic studies to investigate the effect of the magnetic field in the BaSO_4_ precipitation.

Therefore, here we studied the efficiency of the magnetic field on the prevention of barium sulfate incrustation from aqueous solutions. The effect of magnetic field intensity and the field application conditions on precipitation time of this salt is evaluated. An experimental unit was developed based on the dynamic differential pressure technique. This method consists in the injection of solutions through a capillary tube inserted in a furnace for temperature control. Pressure transducers measure the loss of charge along the tube. As a result, scaling time and differential pressure are obtained as a function of different variables such as temperature, pressure, salt concentration, and pH. The results suggest that the magnetic field influences the induction time of the incrustation. Moreover, a semi-empirical model used adequately described the experimental data. X-ray diffraction (XRD) and scanning electron microscopy (SEM) analyses indicate that the magnetic field interferes in the crystalline arrangement and morphology of the precipitated salts.

## 2 Methods

### 2.1 Saline solutions

The solutions used in this study were prepared using barium chloride dihydrate (99%, Synth), sodium chloride (99%, Synth) and sodium sulfate decahydrate (99%, Vetec). While ethylenediaminetetraacetic acid (99%, Synth) and sodium hydroxide (99%, Synth) were used for the preparation of the cleaning solution. Ultrapure water (Millipore 18.2 MΩcm resistance) was used in all solutions. The synthetic saline solution investigated in this work was based on the paper reported by Labraoui-Djallal and Bounoughaz. (2016). The cation solutions contained 542 and 1051 ppm of Na^+^ and Ba^2+^, respectively. The anion solution was prepared using 1300 ppm of SO_4_^−2^. Solutions were prepared by the complete dissolution of the inorganic salts under magnetic stirring (IKA, model RCT basic) for 2 h. The solutions were filtered using a nylon filter of 0.45 μm (Allcrom) coupled to a vacuum pump (Edwards, model RV3). All samples were prepared gravimetrically using an analytical scale (Shimadzu, model AUX220) with an accuracy of 10^−4^ g.

### 2.2 Experimental unit

**Fig. 1:**
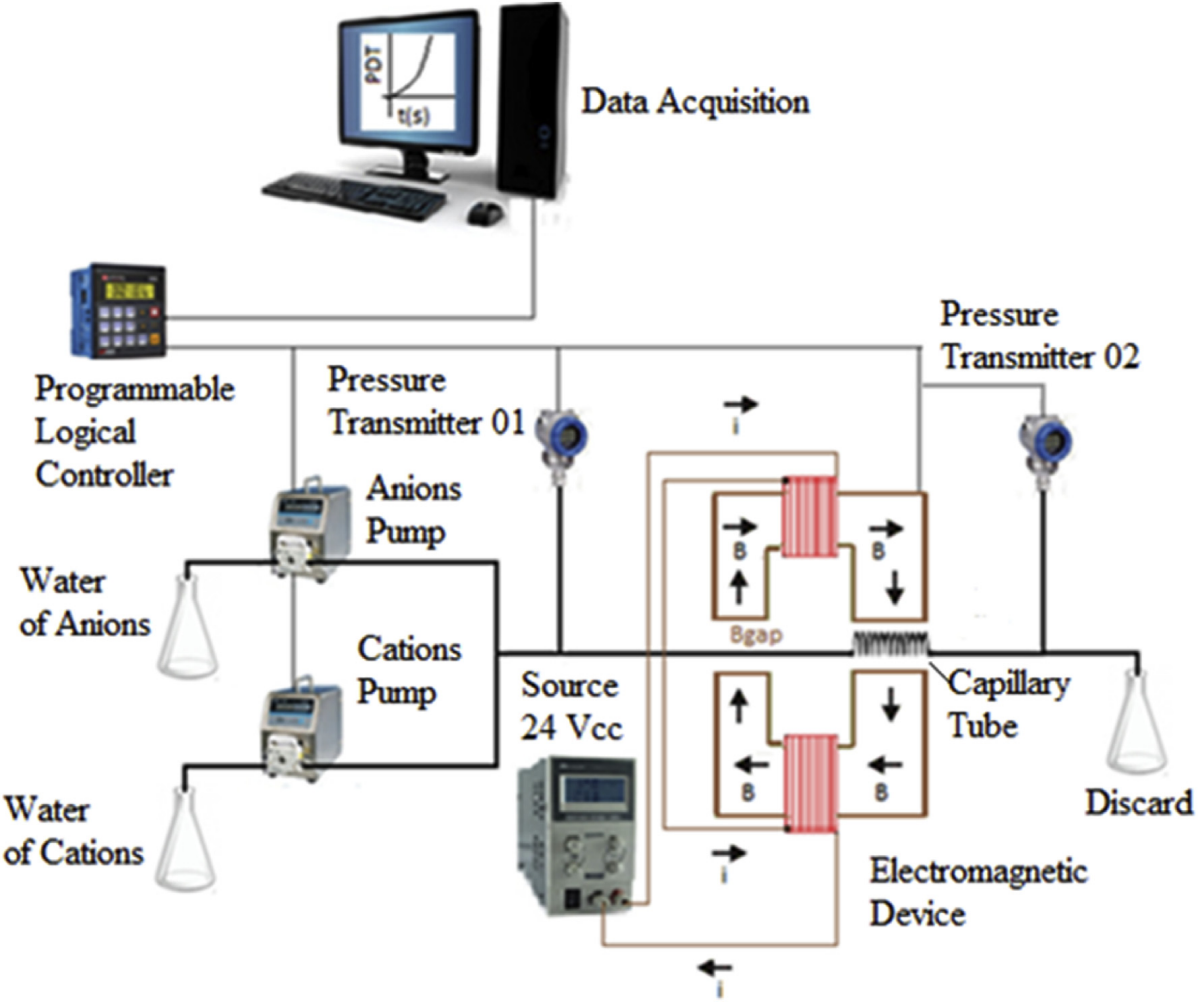
Schematic of the experimental unit used in salt forming assays in the presence of a magnetic field.

A scheme of the experimental unit developed is presented in Fig. 1. The system consists of two positive displacement pumps (Reauxus, model 6010R) with flow rate capacity up to 10 mL min^−1^ and pressures up to 10000 PSI that displace the anion and cation solutions. The same pumps were also used for the displacement of the cleaning solution. The input and output lines, through which the solutions are conducted, are made of stainless steel 1/16" (external diameter) with an internal diameter of 0.045 cm. The data acquisition system consists of two pressure transducers (Ashcroft, model K1, with operating range from 0 to 100 PSI) to monitor the inlet and outlet pressures of the capillary tube, and a programmable logic controller (PLC) (Unitronics Dakol Vision120), coupled to a user interface. Pressure transducers were calibrated with a precision of 0.125% of the full range, and the uncertainty in the pressure measurements was lower than 0.15 PSI. The PLC is the component responsible for the control of both the pumps and the magnetic field, and for the acquisition of pressure data during the performance of the experiments. The Software iFix 4.0 (GE Intelligent Platforms) was used to record and control the variables. The electromagnetic equipment was designed and built to avoid heating, ensuring that the tests occur at room temperature, and guaranteeing the intensity of the magnetic field observed. The system also presents two modes of control of the magnetic field flow: one by the variation of the electric current, by changing the electric potential and another by the variation of the distance between the poles of the electromagnetic system, keeping the intensity of electric current constant.

**Fig. 2:**
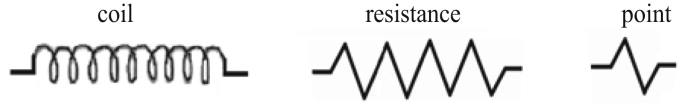
Geometries of capillary tube investigated in barium sulfate precipitation.

In addition, the influence of the type of capillary geometry on barium sulfate incrustation was also investigated. Fig. 2 shows the geometries evaluated, which were called coil (capillary length of 50 cm), resistance (capillary length of 30 cm), and point (capillary length of 10 cm). All the capillary tubes are made of stainless steel (1/16″ of external diameter and 0.045 cm of internal diameter). The initial pressure drop between the inlet and outlet of the devices, measured by the two transducers, was zero (considering the uncertainty of the pressure transducers) for all capillary geometries tested.

### 2.3 Experimental procedure

The experiments were carried out in the absence and the presence of a magnetic field. The two incompatible saline solutions were displaced by HPLC pumps until a mixing point (capillary tube) forming a supersaturated solution. The differential pressure between the beginning and end of the capillary tube was continuously monitored by using the Ifix 4.0 software aiming to determine the time of incrustation. Images of the precipitated crystals were taken by using optical microscopy (ZEISS, model Axiovert 40 MAT). To this end, aliquots were periodically withdrawn during the application of magnetic fields to the saline solutions (until 60 min) and submitted to the optical microscope for analysis. The images were generated using 100 times of magnification.

The analyses of the crystalline structures of samples grown with and without the presence of a magnetic field were performed using XRD measurements. XRD patterns were collected using a multipurpose X-ray diffractometer (Empyrean from PANalytical) with a Cu-K_α_ source (λ = 1.5418 Å) in Bragg–Brentano geometry. The diffraction patterns were recorded at room temperature in the continuous mode with speed 0.25°/min, taken in the angular range 10 < 2θ < 100°. Rietveld refinement analyses were carried out for using the DBWS 9807 code approach as implemented in the DBWSTools2.3 program (Bleicher et al., 2000). Images of SEM were obtained using a JEOL JCM 5700 scanning electron microscope, at a voltage of 5 kV. Magnification ranged from 250 to 9000 times.

All experiments were conducted in duplicate or triplicate, and the statistical analyses were performed in the GraphPad Prism 5.0 program, using analysis of variance (ANOVA), followed by a Tukey test using a confidence interval of 95%.

**Cleaning Procedure.** The cleaning procedure of the experimental unit must ensure the repeatability, reproducibility, and integrity of the data. A 0.1 mol L^−1^ ethylene diamine tetra-acetic solution (Kelland, 2011) was used to remove all barium sulfate crystals contained inside the capillary tube at the end of each experiment. The pH of the solution was adjusted to 10 with the addition of sodium hydroxide. The solution was pumped in the flow of 1.0 mL min^−1^ for 20 min alternating with pure water injection at 2.0 mL min^−1^ for 30 min.

**Magnetic Field.** The Lorentz equation determines the force perceived by a particle or ion in movement (with velocity **v**) in a region of space subjected to the action of a magnetic field, **B**. **B**×**v** is a vector product, and therefore depends on the lag angle that exists between the two vectors as defined by Mosin and Ignatov. (2014). The intensity of this force depends on the module, the direction and the sense of the load speed and the load intensity. The mathematical expression for the intensity of the magnetic force on a moving load is:

(1) $$\vec{F}\;=\;\text{q.}vxB\;\text{or}\;F_{b}=\;\left|q\right|.v.B.sen\theta$$

where $\vec{F}\;$is the magnetic force, q is the module of the electric charge, ***v*** is the load speed, *B* is the magnetic field, and *θ* is the angle between the direction of the load speed and the direction of the magnetic field.

**Semi-empirical model**. The empirical model used for calculating the differential pressure in the tubular pipe was based on the Darcy Weisbach equation (Eq. 2). This model was applied to determine parameters such as the induction time and a reduced growth rate of incrustation. The input data for the calculus were the tube diameter and length, and the differential pressure data collected in the experimental unit, as described by Santos et al. (2017).

(2) $$\Delta P=\frac{32L\rho v^{2}}{Re.d}$$

where Re is the Reynolds number, L is the length of the tube, ρ is the fluid density, v is the kinematic velocity of the fluid and d the internal diameter of the tube. By using the Reynolds number definition and the volumetric flow rate q, the pressure drop equation can be written as:

(3) $$\Delta P=\frac{128\;.\;\;\mu \;.\;\;L\;.\;q\;}{\pi \;.\;D^{4}}$$

The expression for the variation of the tube internal diameter as a function of time is given by:

(4) $$d\left(t\right)=d_{0}-\;\frac{d_{a}}{d_{t}}\;.\;\left(t-t_{ind}\right)\;.\;H\;\left(\;t-t_{ind}\right)$$

where d_0_ is the initial diameter of the tube (tube diameter without precipitated salt), t_ind_ is the incrustation induction time, $\frac{d_{a}}{d_{t}}$ is the incrustation rate. In this simple model, the incrustation rate is considering constant along all the tubing length. H (t–t_ind_) is a function defined as:

(5) $$\text{H}\left(\text{t}\;-{\;\text{t}}_{\text{ind}}\right)=\{\begin{array}{c} 0,\;\;t\leq {\text{t}}_{\text{ind}}\\ 1,\;\;t>{\text{t}}_{\text{ind}} \end{array}$$

The behavior of the salt incrustation in the capillary tube is then provided by the following equation:

(6) $$\Delta \text{P}=\;\frac{128}{\text{π}}\;.\;\frac{\text{μ}\;.\;\text{L}\;.\;\text{q}}{{\left[{\text{d}}_{0}-\frac{{\text{d}}_{\text{a}}}{{\text{d}}_{\text{t}}}\left(\text{t}-{\text{t}}_{\text{ind}}\right).\;\text{H}\left(\text{t}-{\text{t}}_{\text{ind}}\right)\right]}^{4}}$$

The experimental data of the dynamic pressure drop was fitted using the semi-empirical model presented by Eq. (6) estimating two parameters: the induction time (t_ind_) and the incrustation rate $\frac{d_{a}}{d_{t}}$, as presented in detail by Santos et al. (2017). The model and the parameters estimation procedure were implemented in MATLAB 9.4 language. Least square objective function was minimized considering the experimental and model pressure drop along the tube.

## 3 Results and discussion

### 3.1 Effect of the operational variables on the incrustation time without magnetic field application

Table 1 presents the experimental data concerning the incrustation time of barium sulfate in aqueous solutions for the different capillary tube geometries studied. The initial brine solutions investigated were based on the report of Labraoui-Djallal and Bounoughaz. (2016): 1051 ppm of barium cation (7.65 mmol L^−1^ barium chloride dihydrate solution) and 1300 ppm of sulfate anion (13.53 mmol L^−1^ of sodium sulfate decahydrate solution). To evaluate the influence of the salt concentration on the incrustation time, the original brine solution was diluted to produce solutions corresponding to 25%, 50% and 75% of the concentration from the starting solution (263, 525 and 789 ppm of barium ions, and 325, 650 and 975 ppm of sulfate ions, respectively). The total flow rates used were 0.5 and 1.0 mL min^−1^. The Reynolds number and residence time are also illustrated in Table 1.

**Table 1:** Influence of operational variables on the incrustation (induction) time of barium sulfate for different capillary geometries, without using a magnetic field.

Capillary geometry	Concentration from the starting solution (%)	Flow rate (mL·min^−1^)	Reynolds number	Residence time (min)	Incrustation time (min)
Coil	75	1.0	472	0.5	17.6
	75	0.5	236	1.0	34.2
	50	1.0	472	0.5	22.5
	50	1.0	472	0.5	19.7
	50	1.0	472	0.5	23.0
	50	0.5	236	1.0	27.6
	50	0.5	236	1.0	31.3
	25	1.0	472	0.5	36.3
	25	1.0	472	0.5	36.5
Resistance	75	1.0	472	0.3	7.7
	50	1.0	472	0.3	9.7
	50	1.0	472	0.3	9.0
	50	0.5	236	0.6	13.0
	50	0.5	236	0.6	10.0
	25	1.0	472	0.3	19.2
	25	1.0	472	0.3	20.5
	25	0.5	236	0.6	25.2
	25	0.5	236	0.6	23.2
Point	75	1.0	472	0.1	10.2
	75	1.0	472	0.1	10.6
	75	0.5	236	0.2	17.2
	75	0.5	236	0.2	15.7
	50	1.0	472	0.1	11.6
	50	1.0	472	0.1	13.6
	50	0.5	236	0.2	44.5
	50	0.5	236	0.2	37.0
	25	1.0	472	0.1	29.6
	25	1.0	472	0.1	31.4

The incrustation times were selected when the differential pressure between the inlet and outlet pressures reached 3.0 PSI. The incrustation times varied from 7.7 to 45 min. Data in Table 1 were statistically treated by analysis of variance coupled to the Tukey test at a significance level of 95% (p < 0.05). According to data in Table 2, the concentration of 25% of salts presents a significant difference (p < 0.05) on the incrustation time in relation to concentrations of 50% and 75%. In the lowest ions concentration, the supersaturation of the solution is lower and the precipitation of the salts is probably slower, thus increasing the incrustation time.

**Table 2:** Tukey test for the effects of solution concentration, total flow rate and capillary geometry on barium sulfate incrustation time.

Capillary geometry	Concentration	Flow rate
Coil 27.6 + 2.4^A^	75 % of initial 16.2 + 3.3^B^	0.5 mL min^−1^ 25.4 ± 3.3^A^
Resistance 15.3 + 2.2^B^	50 % of initial 21.0 + 3.2^B^	1.0 mL min^−1^ 19.3 ± 2.3^B^
Point 22.1 ± 3.9^A^	25 % of initial 27.7 ± 2.4^A^	

Distinct letters in the same column mean a significant difference (p < 0.05) among the level of variables by using ANOVA and Tukey test.

The type of the device used for the precipitated salts incrustation significantly affects the induction time of barium sulfate incrustation. Hence, the coil is the geometry that presents the longest time for incrustation of the scale in the tests performed. The coil geometry can be considered as a continuous straight line, thus keeping the solution in laminar flow regime. In contrast, both the resistance and the point geometries have specific points where the flow changes its direction and, in this sense, some mixing points are presented.

Additionally, the increase in flow rate increases the velocity of the solutions, but this speed increase was insufficient to change the flow regime, which was considered to be a tubular flow (Table 1). The laminar flow regime was similar to that used by Alimi et al. (2007) in the assessment of both the solubility and the characteristics of CaCO_3_ precipitated in the presence of a magnetic field. On the other hand, by varying the concentration of ions at a constant flow rate, the mass precipitated inside the capillary tube increased, thus decreasing the induction time. Moreover, it should be considered that the point and resistance geometries have some mixing positions that can also help to decrease the incrustation time.

**Fig. 3:**
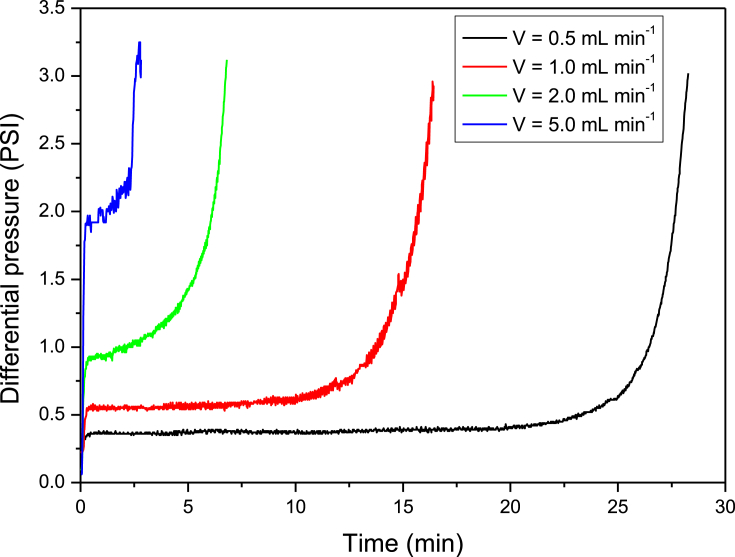
Influence of solution flow rate on the kinetic of barium sulfate incrustation in a resistance capillary tube geometry. Solutions used: 263 mg L^−1^ barium cations and 325 mg L^−1^ sulfate anions.

The resistance geometry was used to evaluate the effect of the flow regime, and the flow rates of anion and cation solutions were increased up to 5.0 mL min^−1^ Fig. 3 shows that the increase in flow velocity decreases the incrustation time, as it promotes more intense shear and contact between particles after formation. Similarly, Saksono et al. (2009) observed that the improvements in the flow increased the precipitation of calcium carbonate particles found in the solution.

### 3.2 Effect of magnetic field on barium sulfate incrustation

**Fig. 4:**
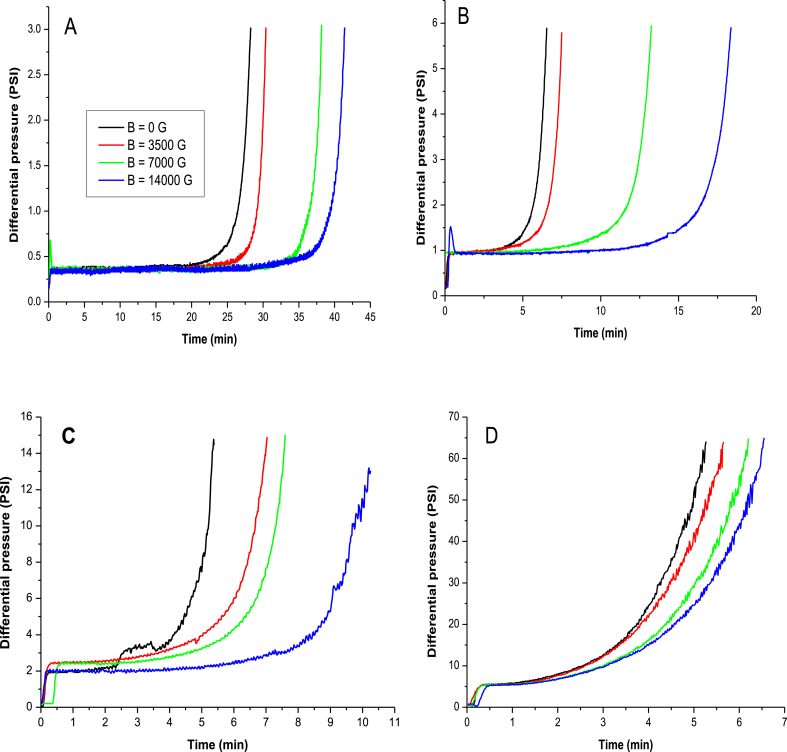
Influence of the magnetic field on the kinetic of barium sulfate incrustation. Concentration of barium cations: 263 mg L^−1^. Concentration of sulfate anions: 325 mg L^−1^. Total flow: 0.5 (A); 2.0 (B); 5.0 (C) and 10 (D) mL min^−1^.

Fig. 4 shows the effect of the magnetic field on the kinetics of the barium sulfate incrustation. The results are depicted for distinct total flow rate in the capillary, from the laminar regime (4A) to a turbulent regime (4D). Magnetic fields up to 14000 G were applied using the resistance capillary geometry. The magnetic field affects the incrustation time for all flow rates studied. The higher the magnetic field, the longer the induction time for the incrustation onset. Consequently, the deposition of the salts in the tube walls is delayed in the presence of the magnetic field. For each total flow rate, as the magnetic field increases, the **B**x**v** product also increases, accordingly preventing the BaSO_4_ incrustation. This effect is due to the increase of the Lorentz force that favors the ion polarization, thus enhancing the ion's hydration as suggested by Kozic et al. (2010), by Silva et al. (2015) and by Al Helal et al. (2018).

Fig. 4 also indicates that as the velocity of the fluid increase, the incrustation takes place at lower times, suggesting that the turbulence and shear of the medium is an important factor in the deposition of the precipitated material. In Fig. 4D, the total flow rate of 10 mL min^−1^ indicates a fluid velocity of 1.04 m s^−1^. When applied a magnetic field of 14000 G, the B×v product results in 14671 G m s^–1^. According to Mosin and Ignatov. (2014) B×v values greater than 10000 G m s^–1^ were necessary to prevent CaCO_3_ incrustation during the treatment of boiler water using a magnetic field. Alternatively, as shown in Table 3, our study suggests that the effect of the magnetic field was clearly observed in much lower B×v products than 10000 G m s^–1^. However, the magnetic field in Fig. 4D is less efficient than in the tests using smaller flow rates (Figure 4A–C), suggesting a strong effect of the flow regime in the incrustation of barium sulfate.

**Table 3:** Influence of the magnetic field and the total flow rate on the barium sulfate incrustation time in a resistance geometry. Concentration of barium cations: 263 mg L^-1^. Concentration of sulfate anions: 325 mg L^-1^.

Reynolds Number	Flow rate (ml min^−1^)	Magnetic Field (Gauss)	Bˆv (Gauss m s^−1^)	Incrustation time (min)
236	0.5	0	0	28.3
236	0.5	3750	196.5	30.4
236	0.5	7000	366.8	36.0
236	0.5	14000	733.6	41.6
944	2	0	0	6.5
944	2	3750	786	7.5
944	2	7000	1467.1	13.2
944	2	14000	2934.2	18.3
2359	5	0	0	5.3
2359	5	3750	1964.9	6.9
2359	5	7000	3667.8	7.5
2359	5	14000	7335.5	10.2
4718	10	0	0	5.3
4718	10	3750	3929.8	5.7
4718	10	7000	7335.5	6.2
4718	10	14000	14671.1	6.6

These findings are quantitatively evidenced in Table 3, which shows the effect of the fluid velocity and the magnetic field intensity on the incrustation time of the BaSO_4_. As the fluid velocity increased, the incrustation time decreased, as seen in Fig. 3, whereas the magnetic field intensity tends to enhance the incrustation time (Fig. 4).

### 3.3 Semi-empirical modeling

**Fig. 5:**
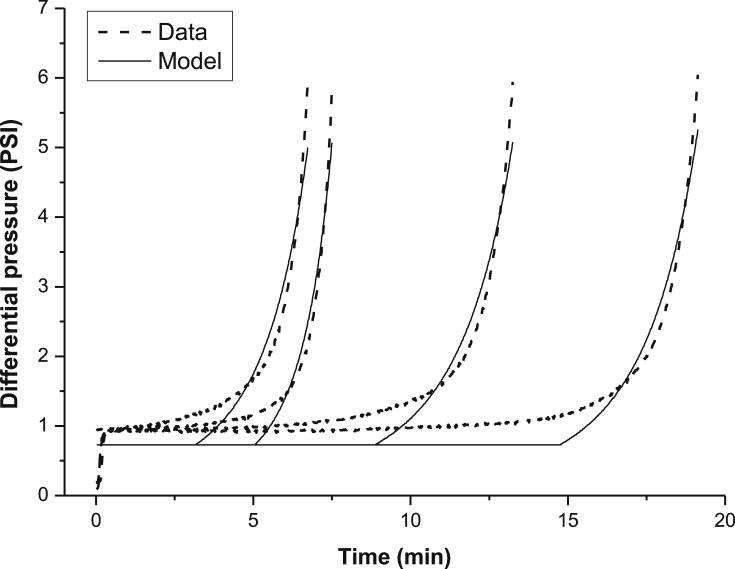
Description of the dynamics of the pressure drop during the barium sulfate incrustation by the semi-empirical model. Concentration of barium cations: 263 mg L^−1^. Concentration of sulfate anions: 325 mg L^−1^. Total flow: 2.0 mL min^−1^.

Fig. 5 presents the performance of the semi-empirical model in the description of the BaSO_4_ incrustation kinetics employing Eq. (6). The experimental data refer to barium cations concentration of 263 mg L^−1^ and 325 mg L^−1^ of sulfate anions, with a total flow rate of 2.0 mL min^−1^ and diverse intensities of the magnetic field applied. The results indicate that the model can reproduce the kinetics of pressure drop during barium sulfate incrustation.

Table 4 shows the effects of the processing variables on the incrustation rate and on the incrustation time parameters from the semi-empirical model. Notably, the higher the flow rate, or the fluid velocity, the lower the incrustation time, due to the higher shear of the solution. On the other hand, the magnetic field tends to smooth this effect increasing the incrustation time. The application of a magnetic field with high intensity leads to a decrease in the incrustation rate, suggesting that the decrease rate of the tube diameter by the particle deposition is slower when the magnetic field is applied.

**Table 4:** Semi-empirical model parameters obtained from the dynamic test modeling of the barium sulfate incrustation in the presence of a magnetic field. Concentration of barium cations: 263 mg L^-1^ and 325 mg L^-1^ of sulfate anions.

Magnetic Field (Gauss)	Flow Rate (mL min^−1^)	B×v product (Gauss.m.s^−1^)	Incrustation rate (microns min^−1^)	Incrustation time (min)
0	0.5	0	1959	25.2
3500	0.5	196	1.341	28.3
7000	0.5	367	1400	35.3
14000	0.5	734	1214	38.0
0	1.0	0	1222	11.3
3500	1.0	367	1004	12.9
7000	1.0	734	910	17.7
14000	1.0	1467	831	19.9
0	2.0	0	1931	4.3
3500	2.0	786	1743	5.1
7000	2.0	1467	975	8.9
14000	2.0	2934	791	13.0

**Fig. 6:**
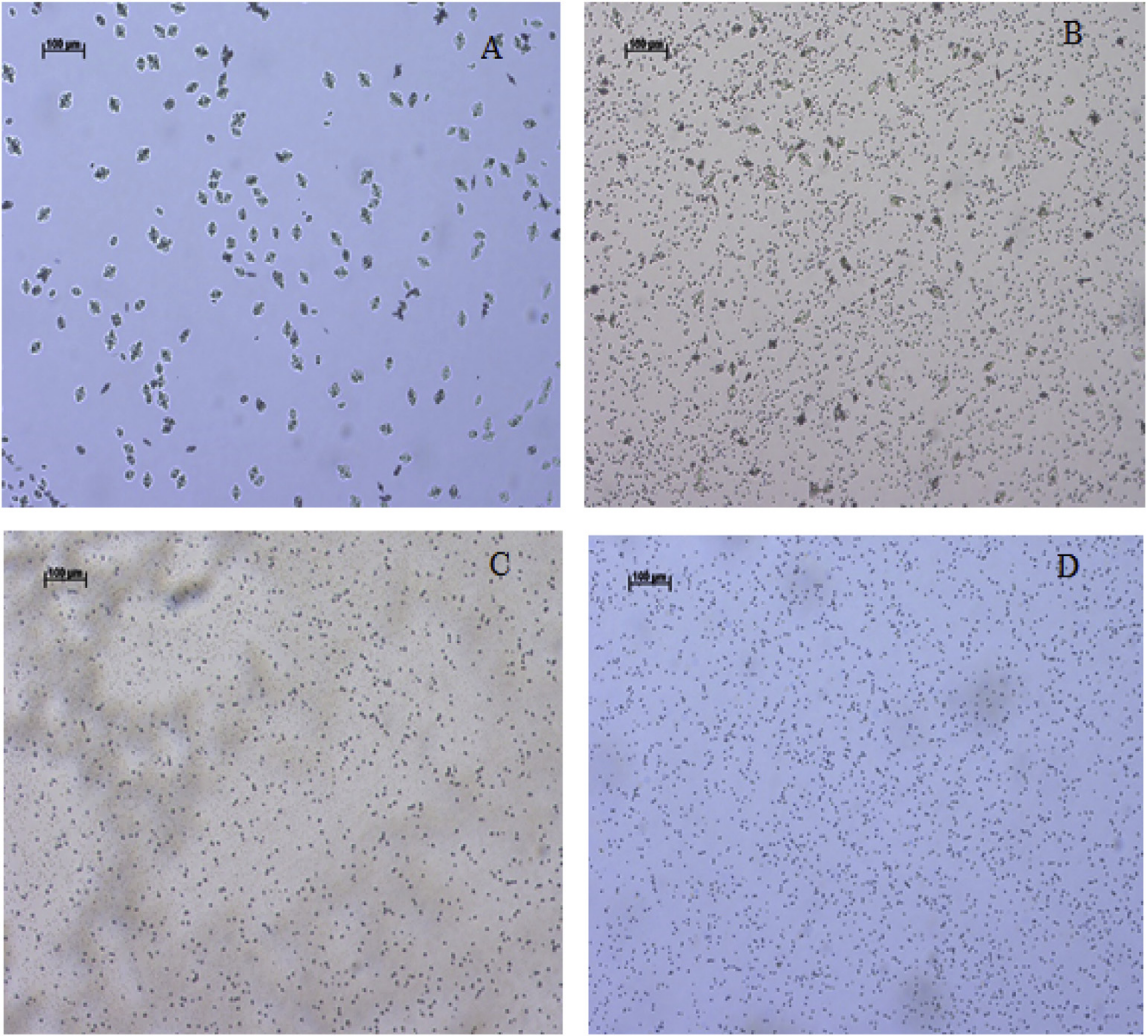
Optical microscopy images, taken for barium sulfate particles produced with the application of distinct magnetic fields. (A) 0 G (B) 3500 G (C) 7000 G and (D) 14000 G. Images taken after 60 min of the experiment.

Fig. 6 displays images obtained by optical microscopy depicting precipitated BaSO_4_ particles in the presence of distinct magnetic field intensities, applied for 60 min. The images show that the presence of the magnetic field increases the number of precipitated particles. Nevertheless, the crystals formed are smaller when compared with the sample obtained without applied magnetic field at the same time of analysis. In addition, the size of the precipitated particles seems to decrease with increasing of the magnetic field intensity. This finding corroborates the diminishing in the incrustation rate and the enhancement in the incrustation time, as the smaller particles induced by the magnetic field delays the growth of the crystals in the tube walls.

**Fig. 7:**
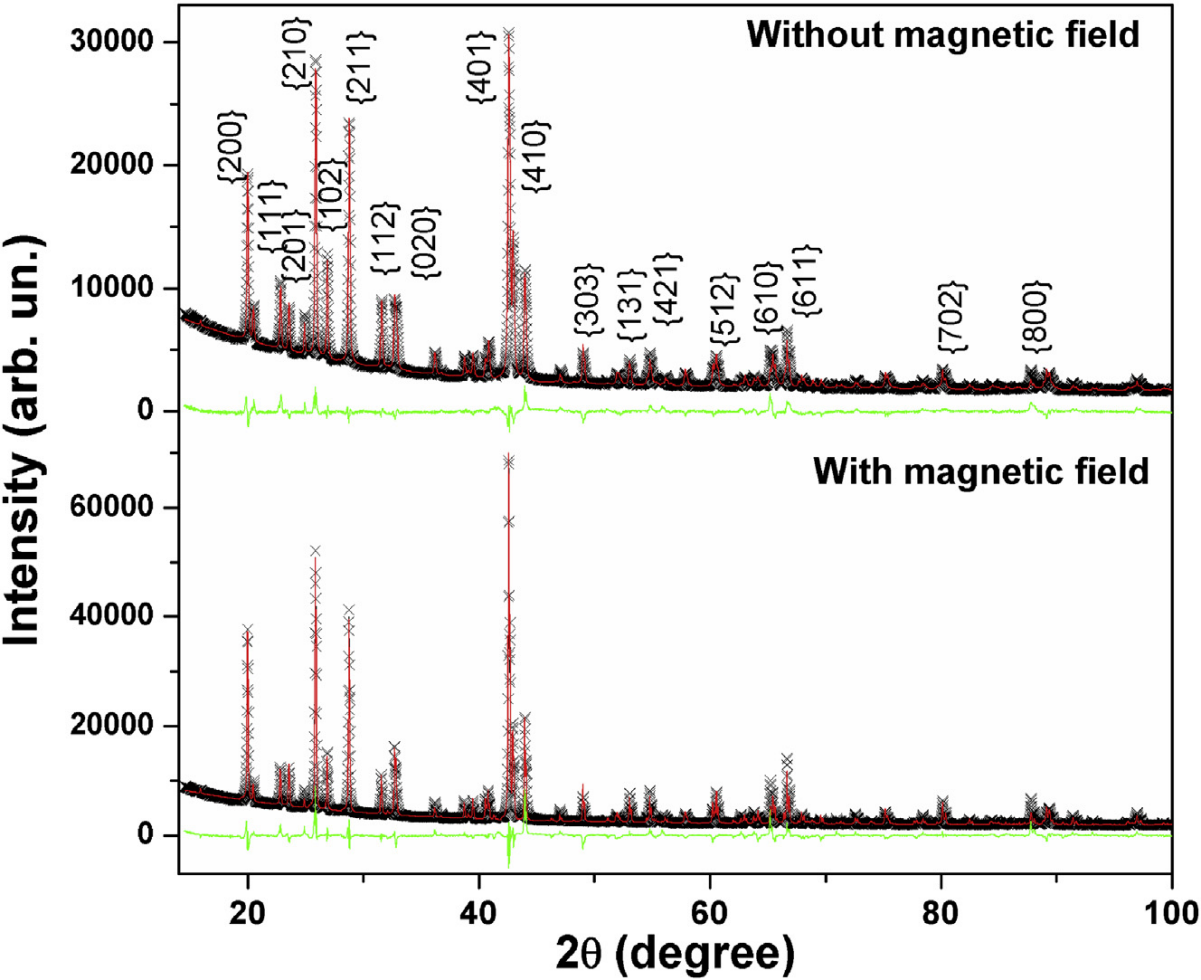
XRD patterns of the barium sulfate precipitated in the presence and the absence of magnetic field.

The analysis of XRD patterns (Fig. 7) allows us to corroborate the observations from microscopy images (Fig. 6). The crystals obtained with magnetic field application present crystallite size slightly smaller and greater crystallinity than those formed without the application of the magnetic field. This behavior indicates a slower growth velocity in the presence of the magnetic field, generating a more ordered system.

Additionally, the Rietveld refinements considering the tetragonal phase with P*nma* space group confirm the formation of crystalline barium sulfate. From these refinements, the calculated BaSO_4_ phase amount was of 100 mol% for both samples, whose structural refined parameters are summarized in Table 5. These parameters are in good agreement with reported data for the same BaSO_4_ composition at ambient condition (Crichton et al., 2011). Moreover, Rietveld refinements using a preferred orientation axis and refining the March–Dollase coefficient for Bragg peak family {401}, which was improved for all fits, shows a tendency orientation of this plane for two samples. Therefore the sample prepared with magnetic field present an orientation degree 10% higher than the sample obtained without magnetic field when the values are estimated by the Lotgering method (Lotgering, 1959).

**Table 5:** Refined structural parameters of BaSO4 samples treated with and without field using tetragonal structure (space group Pnma).

Ions	Site	With Field			Without Field		
		X	y	Z	x	y	z
Ba^2+^	4c	0.1849 (1)	0.2500	0.1476 (3)	0.1848 (1)	0.2500	0.1574 (3)
S^6+^	4c	0.0672 (8)	0.2500	0.688 (2)	0.0709 (6)	0.2500	0.7029 (1)
O^2–^(I)	4c	-.0773 (8)	0.2500	0.600 (2)	-.0783 (6)	0.2500	0.600 (2)
O^2–^(II)	4c	0.1770 (8)	0.2500	0.480 (1)	0.1756 (9)	0.2500	0.525 (2)
O^2–^(III)	8d	0.0910 (6)	0.0769 (3)	0.778 (2)	0.0865 (5)	0.0299 (2)	0.779 (1)
		Crystallite size = 74 (5) nm R_wp_ = 7.85% $\chi^{2}$ = 4.36 *a* = 8.8867 (1) Å *b* = 5.4520 (1) Å *c* = 7.1528 (2) Å V = 346.56 (1) Å^3^			Crystallite size = 100 (10) nm R_wp_ = 4.81% $\chi^{2}$ = 2.41 *a* = 8.8883 (1) Å *b* = 5.4465 (2) Å *c* = 7.1494 (2) Å V = 346.10 (2) Å^3^		

**Fig. 8:**
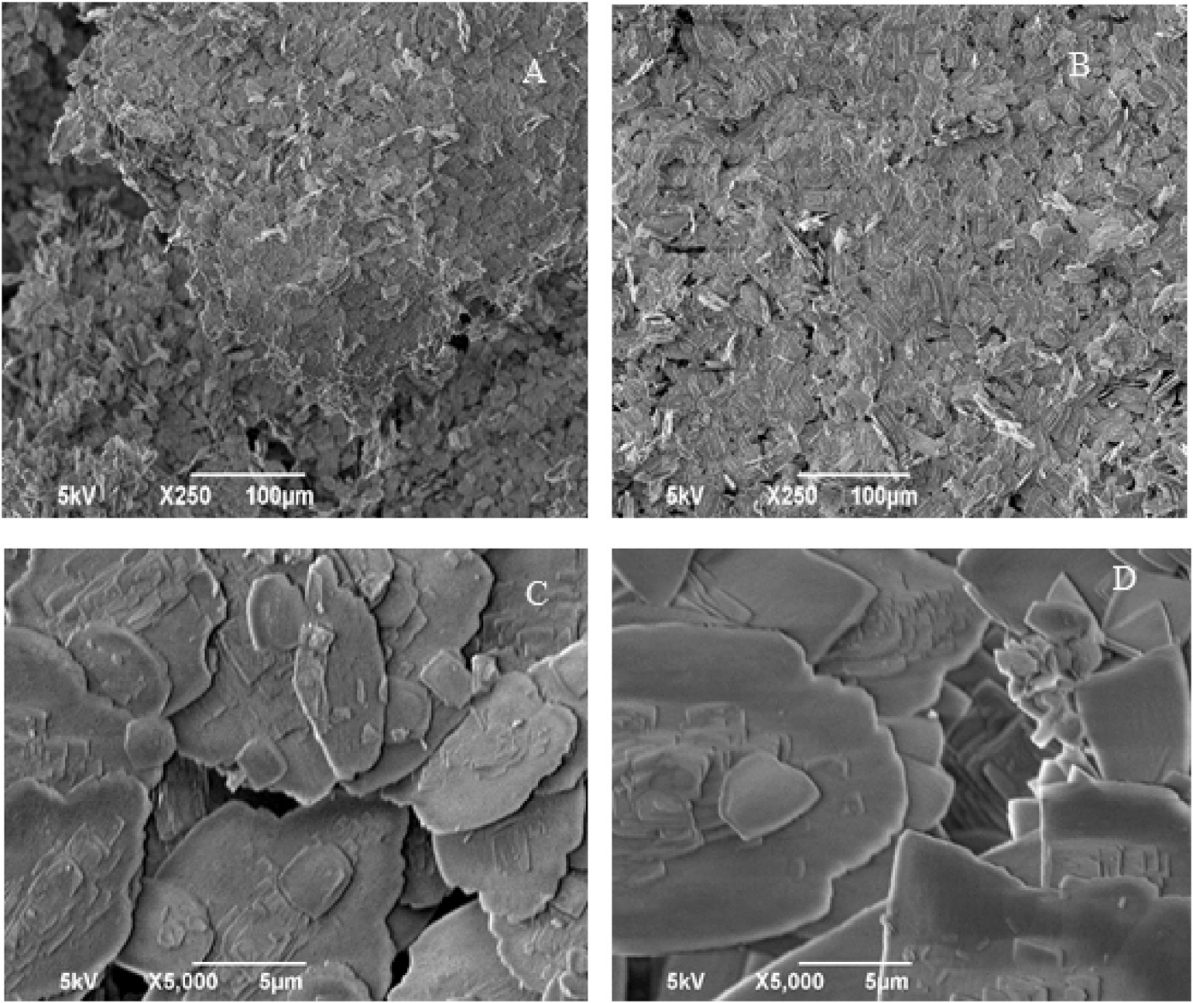
SEM patterns of barium sulfate particles precipitated in the absence of field (A e C) and the presence (B e D) of the magnetic field (intensity of 14000 G).

Fig. 8 shows SEM images taken for the precipitated BaSO_4_ samples treated without and with a magnetic field of 14000 G. A slightly different morphology is observed for the crystals precipitated in the presence of the magnetic field, which are somewhat more arranged and with more defined crystals, supporting the greater crystallinity observed in Fig. 7.

## 4 Conclusions

In this work, an experimental unit based on the dynamic differential pressure monitoring was developed to study the BaSO_4_ incrustation in the presence of different magnetic field intensities. The results suggested that the magnetic field application decreases the velocity of growth of barium sulfate particles, resulting in larger incrustation times. The barium sulfate particles grown in the presence of magnetic field presented greater crystallinity and smaller crystalline size than those formed without field. In addition, the application of magnetic fields in samples with barium cations and sulfate anions delays the induction time of barium sulfate incrustation. Consequently, the application of magnetic fields can be a potential tool to combat the formation of this salt incrusted in pipelines of the petroleum industry.

## Declarations

### Author contribution statement

Zurel Costa: Conceived and designed the experiments; Performed the experiments; Analyzed and interpreted the data; Wrote the paper.

Cristiano Meneses, Fabiane S. Serpa, Elton Franceschi, Gustavo Borges: Analyzed and interpreted the data.

Bruno Castro: Contributed reagents, materials, analysis tools or data.

Claudio Dariva, Giancarlo Salazar-Banda: Conceived and designed the experiments; Analyzed and interpreted the data; Wrote the paper.

### Funding statement

This work was supported by the CNPq (grants 304419/2015–0 and 305438/2018–2), to the Coordenação de Aperfeiçoamento de Pessoal de Nível Superior - CAPES (Grant: 001) and to FAPITEC/SE from Brazil.

### Competing interest statement

The authors declare no conflict of interest.

### Additional information

No additional information is available for this paper.

## References

[bib1] Al Helal A., Soames A., Gubner R., Iglauer S., Barifcani A. (2018). Influence of magnetic fields on calcium carbonate scaling in aqueous solutions at 150°C and 1 bar. J. Colloid Interface Sci..

[bib2] Al Helal A., Soames A., Iglauer S., Gubner R., Barifcani A. (2019). The influence of magnetic fields on calcium carbonate scale formation within monoethylene glycol solutions at regeneration conditions. J. Pet. Sci. Eng..

[bib3] Alimi F., Tlili M., Ben Amor M., Gabrielli C., Maurin G. (2007). Influence of magnetic field on calcium carbonate precipitation. Desalination.

[bib4] Antony A., Low H.J., Gray S., Childress A.E., Le-Clech P., Leslie G. (2011). Scale formation and control in high pressure membrane water treatment systems: a review. J. Membr. Sci..

[bib5] Bezerra C.M., Rosario F.F., Rosa K.R.S.A. (2013). Scale Management in Deep and Ultradeep Water fields.

[bib6] BinMerdhah A.B., Yassin A.M., Muherei M.A. (2010). Laboratory and prediction of barium sulfate scaling at high-barium formation water. J. Pet. Sci. Eng..

[bib7] Bleicher L., Sasaki J.M., Santos C.O.P. (2000). Development of a graphical interface for the Rietveld refinement program DBWS. J. Appl. Crystallogr..

[bib8] Cefalas A.C., Kobe S., Drazic G., Sarantopoulou E., Kollia Z., Strazisar J., Meden A. (2008). Nanocrystallization of CaCO_3_ at solid/liquid interfaces in magnetic field: a quantum approach. Appl. Surf. Sci..

[bib9] Chang M., Tai C.Y. (2010). Effect of the magnetic field on the growth rate of aragonite and the precipitation of CaCO_3_. Chem. Eng. J..

[bib10] Chibowski E., Szcześ A. (2018). Magnetic water treatment – a review of the latest approaches. Chemosphere.

[bib11] Crichton W.A., Merlini M., Hanfland M., Muller H. (2011). The crystal structure of barite, BaSO_4_, at high pressure. Am. Mineral..

[bib14] Fan C., Kan A.T., Tomson M.B. (2009). Barite Nucleation and Inhibition at 0–200°C with and without Hydrate Inhibitors:.

[bib16] Jiang L., Zhang J., Li D. (2013). Effects of permanent magnetic field on calcium carbonate scaling of circulating water. Desalin. Water Treat..

[bib18] Jordan M.M., Williams H., Linares-Samaniego S., Frigo D.M. (2014). New Insights on the Impact of High Temperature Conditions (176°C) on Carbonate and Sulphate Scale Dissolver Performance.

[bib19] Kelland M.A. (2011). Effect of various cations on the formation of calcium carbonate and barium sulfate scale with and without scale inhibitors. Ind. Eng. Chem. Res*.*.

[bib20] Khormali A., Sharifov A.R., Torba D.I. (2018). Increasing efficiency of calcium sulfate scale prevention using a new mixture of phosphonate scale inhibitors during waterflooding. J. Pet. Sci. Eng..

[bib21] Kim H.N., Suslick K.S. (2018). The effects of ultrasound on crystals: sonocrystallization and sonofragmentation. Crystals.

[bib22] Kozic V., Hamler A., Ban I., Lipus L.C. (2010). Magnetic water treatment for scale control in heating and alkaline conditions. Desalin. Water Treat..

[bib23] Labraoui-Djallal K.L., Bounoughaz M. (2016). Evaluation efficiency of barium sulfate scale inhibitors by electrochemical impedance spectroscopy. Int. J. Electrochem. Sci..

[bib24] Lotgering F.K. (1959). Topotactical reactions with ferrimagnetic oxides having hexagonal crystal structures-I. J. Inorg. Nucl. Chem..

[bib25] Mosin O., Ignatov I. (2014). Basic concepts of magnetic water treatment. Nanotechnol. Res. Pract..

[bib26] Reis M.I.P., Silva F.C., Romeiro G.A., Rocha A.A., Ferreira V.F. (2011). Deposição Mineral em Superfícies: problemas e Oportunidades na Indústria do Petróleo. Rev. Virtual Quim..

[bib27] Rosa K.R.S.A., Bezerra M.C.M., Fontes R.A. (2016). Study of thermal stability of scale inhibitors and its impact on processing plants. International Oilfield Scale Conference and Exhibition.

[bib28] Saksono N., Yuliusman Y., Bismo S., Soemantojo R., Manaf A. (2009). Effects of pH on calcium carbonate precipitation under magnetic field. Makara J. Technol..

[bib29] Santos H.F.L., Castro B.B., Bloch M., Martins A.L., Schlüter H.E.P., Júnior M.F.S., Jacinto C.M.C., Rosário F.F. (2017). A Physical Model for Scale Growth during the Dynamic Tube Blocking Test..

[bib31] Silva I.B., Neto J.C.Q., Petri D.F.S. (2015). The effect of magnetic field on ion hydration and sulfate scale formation. Colloids Surf., A.

[bib32] Simonic M., Urbancl D. (2017). Alternating magnetic field influence on scaling in pump diffusers. J. Clean. Prod..

[bib33] Sohaili J., Shi H.S., Baloo L., Zardari N.H., Ahmad N., Muniyandi S.K. (2016). Removal of scale deposition on pipe walls by using magnetic field treatment and the effects of magnetic strength. J. Clean. Prod..

[bib34] Vazirian M.M., Charpentier T.V.J., Penna M.O., Neville A. (2016). Surface inorganic scale formation in oil and gas industry: as adhesion and deposition processes. J. Pet. Sci. Eng..

[bib35] Wang S.S.S., Chang M.-C., Chang H.C., Chang M.H., Tai C.Y. (2012). Growth behavior of aragonite under the influence of magnetic field. Temperature. And impurity. Ind. Eng. Chem. Res..

[bib36] Zendehboudi S., Shafiei A., Bahadori A., James L.A., Elkamel A., Lohi A. (2014). Asphaltene precipitation and deposition in oil reservoirs – technical aspects. experimental and hybrid neural network predictive tools. Chem. Eng. Res. Des..

